# Effects of a rapid response system on quality of life: a prospective cohort study in surgical patients before and after implementing a rapid response system

**DOI:** 10.1186/1477-7525-11-74

**Published:** 2013-05-01

**Authors:** Friede Simmes, Lisette Schoonhoven, Joke Mintjes, Bernard G Fikkers, Johannes G van der Hoeven

**Affiliations:** 1 Faculty of Health and Social Studies, HAN University of Applied Sciences, PO Box 6960, Nijmegen, 6503 GL, Netherlands; 2 Scientific Institute for Quality of Healthcare Radboud University Nijmegen Medical Centre, Nijmegen, Netherlands; 3 Faculty of Health Sciences, University of Southampton, Southampton, UK; 4 Department of Intensive Care Medicine, Radboud University Nijmegen Medical Centre, Nijmegen, Netherlands

**Keywords:** Hospital rapid response team, Medical emergency team, Quality of life, EuroQol, General surgery

## Abstract

**Background:**

The aim of a rapid response system (RRS) is to improve the timely recognition and treatment of ward patients with deteriorating vital signs The system is based on a set of clinical criteria that are used to assess patient’s vital signs on a general ward. Once a patient is evaluated as critical, a medical emergency team is activated to more thoroughly assess the patient’s physical condition and to initiate treatment. The medical emergency team included a critical care physician and a critical care nurse.

**Aim:**

To assess the effect of an RRS on health-related quality of life (HRQOL).

**Methods:**

Prospective cohort study in surgical patients before and after implementing an RRS. HRQOL was measured using the EuroQol-5 dimensions (EQ-5D) and the EQ visual analogue scale (VAS) at pre surgery and at 3 and 6 months following surgery.

**Results:**

No statistical significant effects of RRS implementation on the EQ-5D index and EQ-VAS were found. This was also true for the subpopulation of patients with an unplanned intensive care unit admission. Regarding the EQ-5D dimensions, deterioration in the ‘mobility’ and ‘usual activities’ dimensions in the post-implementation group was significantly less compared to the pre-implementation group with a respective mean difference of 0.08 (p = 0.03) and 0.09 (p = 0.04) on a three-point scale at 6 months. Lower pre-surgery EQ-5D index and higher American Society of Anesthesiologists physical status (ASA-PS) scores were significantly associated with lower EQ-5D index scores at 3 and 6 months following surgery.

**Conclusions:**

Implementation of an RRS did not convincingly affect HRQOL following major surgery. We question if HRQOL is an adequate measure to assess the influence of an RRS. Pre-surgery HRQOL- and ASA-PS scores were strongly associated with HRQOL outcomes and may have abated the influence of the RRS implementation.

## Background

Rapid response systems (RRSs) are considered a powerful tool in patient safety. The aim of an RRS is to improve the timely recognition and treatment of general ward patients with deteriorating vital signs. The system is based on a set of clinical criteria that are used to assess patient’s vital signs on a general ward. Once a patient’s status is evaluated as critical according to these criteria [1], a rapid response team is activated to more thoroughly assess the patient’s physical condition and to initiate treatment [2].

The most frequently used outcome measure to evaluate the effectiveness of an RRS is the incidence of serious adverse events (SAEs), including cardiac arrest rate, (unexpected) death and unplanned intensive care unit (ICU) admission [3-6]. Previously, we showed that the introduction of an RRS on a surgical ward resulted in a statistically non- significant decrease in patients who experienced a cardiac arrest and/or who died unexpectedly on the ward while unplanned ICU admissions of patients increased significantly [7]. In addition to these medical outcomes, quality of life measures are also becoming increasingly important to health care research. Quality of life outcomes reflect a patient’s health perspective and are relevant to better understand and improve healthcare expenditure and resource utilisation in patient care [8]. We hypothesised that the RRS system would positively influence patient’s quality of life. The aim of the current study was to estimate the effect of an RRS on the quality of life at 3 and 6 months following surgery in the entire study population and in the subset of patients with an unplanned ICU admission.

## Methods

We measured health-related quality of life (HRQOL) at pre-surgery and at 3 and 6 months following surgery in patients admitted to the surgical ward of a university hospital. Measurements were taken over two 12-months periods. Period 1 was conducted before the implementation of an RRS from January 2006 until December 2006. Period 2 was conducted after introduction of an RSS from April 2007 until April 2008. The local medical ethics committee waived the need for informed consent.

In our study we included patients staying on the surgical ward ≥72 hours because of major general surgery, including central or extensive peripheral vascular surgery, major oncologic surgery, lung surgery, extensive abdominal surgery and trauma. The 72-hours limit was used to exclude patients with minor surgical procedures. Patients unable to communicate effectively were also excluded. In period 1, a convenience sample of 518 of 1376 eligible patients were screened for participation and in period 2, 549 of 2410 patients.

HRQOL was measured using the Euroqol 5 dimensions (EQ-5D) and Euroqol visual analogue scale (EQ-VAS) questionnaire, an extensively validated instrument and approved by the Euroqol Translation Committee [9]. EQ-5D measures the following health dimensions: mobility, self-care, usual activities, pain/discomfort and anxiety/depression. Each dimension is divided into three levels: level 1 = no problems, level 2 = some/moderate problems, level 3 = severe/ extreme problems. The EQ-5D index values are derived from a general Dutch population sample [10] and range from minus 0.33 to plus 1. The EQ-VAS measures overall health on a scale from 0 to 100.

In addition, socio-demographic and clinical variables influencing HRQOL were recorded. These included age, sex, education level, employment status and smoking behaviour [11,12]. We also recorded the length of stay (LOS) of planned and unplanned ICU admissions and the American Society of Anaesthesiologists physical status (ASA-PS) classifications score at ICU admission.

The RRS system was introduced in January 2007 and was fully operational by April 2007. The system required ward nurses to systematically observe and record patient’s vital signs at least three times daily. If nurses felt worried about a patient’s condition or observed abnormal vital indicators, then they were instructed to immediately call the ward physician. Abnormal vital indicators included respiratory rate <8 or >30 per minute, oxygen saturation <90%, systolic blood pressure <90 or >200 mm Hg, heart rate <40 or >130 per minute, and a decrease of two points in the eye, motor, and verbal (EMV) score [13]. Once called, the ward physician was required to evaluate the patient at bedside within 10 minutes and to immediately call the medical emergency team (MET) if the patient’s condition was serious or if the patient did not stabilise after an initial intervention. The MET included a critical care physician and a critical care nurse. If the ward physician could not see the patient within 10 minutes, nurses were instructed to activate the MET directly.

### Data collection

Eligible patients were approached on the surgical ward before surgery, or in the case of emergency surgery, immediately after surgery. The research assistant explained the study objectives orally and in writing. Participating patients were asked to fill in the EQ-5D and EQ-VAS based on their condition the day before hospital admission. Patients were also asked to fill in the questionnaires at 3 and 6 months after surgery. Non-responders were contacted twice. Additional clinical variables were retrieved from the hospital’s electronic databases.

### Statistical analysis

Normally distributed data were parametrically tested with the independent Student’s t test, non-normally distributed data with the Mann–Whitney U test, and nominal data with the chi-square test. Differences in HRQOL outcomes in period 1 and 2 were tested with the analysis of covariance (ANCOVA). At Pre- surgery the fixed factors ‘gender’, ‘ASA-PS’ and the covariate ‘age at admission’ were used. At the 3- and 6-month follow-up, the covariates ‘EQ-5D pre-surgery’ or ‘EQ-VAS pre-surgery’ and ‘planned ICU LOS’ were also used. In addition, we compared HRQOL in period 1 and 2 in a subset of patients with unplanned ICU admissions. For statistical analysis, the statistical package for the social sciences (SPSS) version 17 was used. In our analysis a p < 0.05 was considered statistically significant.

## Results

**Figure 1:**
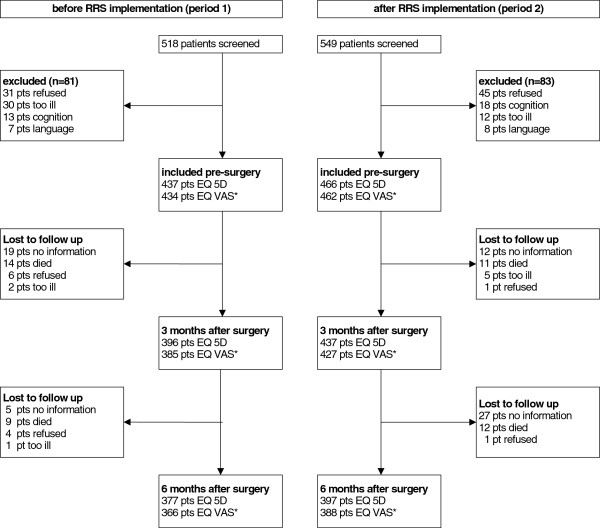
**Overview of included surgical patients.** RRS = rapid response system; before = before implementing the RRS; after = after implementing the RRS; EQ-5D = Euroqol 5 dimensions; EQ VAS = Euroqol visual analogical scale; pts = patients. *Not all patients filled in the VAS score.

In period 1, 84% (437/518) of the screened patients were included in the study, while in period 2, 85% (466/549) of the screened patients were included (Figure 1). Table 1 shows the characteristics of the in- and excluded patients. Excluded patients were not significantly different from included patients regarding gender or age. However, the ASA-PS score of excluded patients was 0.3 points (p < 0.001) higher in both periods. Demographics for the final study group are shown in Table 2. Patients lost to follow up were significantly younger: 6 years *(p = 0.05)* in period 1, and 8 years (*p ≤ 0.01*) in period 2.

**Table 1:** Characteristics of excluded and included patients

	**Excluded**	**Included**	**p-value**
**Before RRS implementation**	n = 81	n = 437	
Gender male (%)	40 (49)	225 (52)	0.58
Mean age mean (SD)	57 (21)	56 (15)	0.41
ASA-PS (SD)	2.3 (0.9)	2.0 (0.8)	0.01
**After RRS implementation**	n = 83	n = 466	
Gender male (%)	42 (51)	239 (51)	0.83
Age mean (SD)	61 (18)	58 (16)	0.07
ASA-PS (SD)	2.4 (0.8)	2.1 (0.7)	<0.01

RRS = rapid response system; SD = standard deviation; ASA-PS = American Society of Anesthesiologists physical status.

**Table 2:** Characteristics of included patients

	**Before n = 437**		**After n = 466**		**p-value**
Gender male (%)	225	(51.5)	239	(51.3)	0.95
Mean age mean SD)	56.1	(15.3)	57.8	(16.2)	0.37
ASA PS mean (SD)	2.03	(0.8)	2.08	(0.7	0.16
Unemployed (%)	6	(1.4)	8	(1.7)	0.54
Education, low level (%)	46	(10.9)	62	(13.3)	0.28
Smoking (%)	70	(16.3)	77	(16.6)	0.92

Before = before implementing the rapid response system (RRS); after = after implementing the RRS; ASA PS = American Society of Anesthesiologists physical status; SD = standard deviation.

### Effects of RRS implementation on quality of life

**Figure 2:**
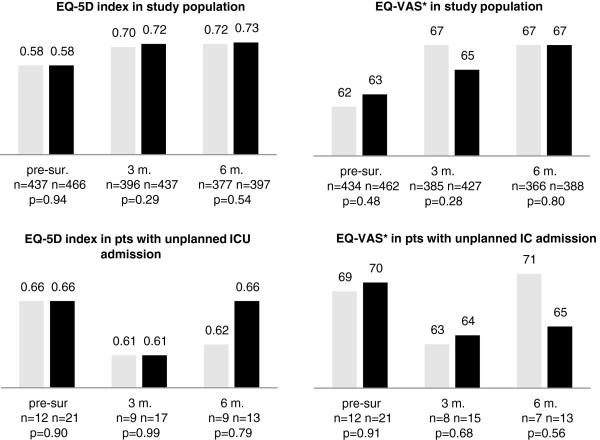
**EQ-5D and VAS mean scores of surgical patients.** Grey: period 1 = before implementing the rapid response system; black: period 2 = after implementing the rapid response system; 3 m. = 3months after surgery, 6m. = 6 months after surgery; EQ-5D = Euroqol 5, scale -0.33 - 1; VAS = visual analogue scale 0–100; RRS = rapid response system. Pre-surgery: fixed factors: gender, American Society of Anaesthesiologists’ physical status (ASA-PS), covariates: age at admission. Following surgery: fixed factors: gender, ASA-PS, covariates: age at admission, planned intensive care length of stay not because of a serious adverse event, EQ-5D dimension pre-surgery. *Not all patients filled in the VAS score.

Figure 2 shows the results of RRS implementation on the quality of life. In both period 1 and 2 patients’ HRQOL was improved at 3 and 6 months following surgery. When we compared period 1 and 2, there were no statistical differences in either the EQ-5D index (0.72 versus 0.73, *p = 0.54* at 3 months following surgery and 0.70 versus 0.72, *p = 0.29* at 6 months following surgery) or the EQ-VAS scores (67 versus 65*, p = 0.28* at 3 months following surgery and 67 versus 67, *p = 0.80* at 6 months following surgery). This was also true for patients with an unplanned ICU admission. HRQOL, however, decreased at 3 months and was near pre-surgery level at 6 months following surgery. In this subset of patients the EQ-5D index was 0.61 versus 0.61, *p = 0.99* at 3 months following surgery and 0.62 versus 0.66, *p = 0.79* at 6 months following surgery while the EQ-VAS was 69 versus 70, *p = 0.91* at 3 months following surgery and 71 versus 65, *p = 0.56* at 6 months following surgery.

#### EQ-5D dimensions

Results of the EQ-5D dimensions are shown in Table 3. In both period 1 and 2, patients reported fewer problems on the EQ dimensions ‘pain/discomfort’ and ‘anxiety/depression’ but more problems with ‘mobility’, ‘self-care’ and ‘usual activities’ at 3 and 6 months following surgery. In period 2 at 6 months, however, patients experienced slightly less deterioration regarding ‘mobility’ and ‘usual activities’ than they did in period 1 (mean difference between period 1 and 2 was 0.08, *p = 0.03* for ‘mobility’ and 0.09, *p = 0.04* for ‘usual activities’ on a 3 point scale).

**Table 3:** EQ-5D dimensions of surgical patients

	**Before**		**After**		**Differences**		
	**n**	**mean**	**n**	**mean**	**of mean**	**95% CI**	***p-value***
**Mobility**							
Pre-surgery	437	1.57	466	1.53	0.04	-0.43 - 0.12	*0.36*
3 months after surgery	396	1.76	437	1.73	0.04	-0.04 - 0.10	*0.28*
6 months after surgery	377	1.79	397	1.72	0.08	0.01 - 0.14	0.03*
**Self-care**							
Pre-surgery	437	1.26	466	1.25	0.02	-0.05 - 0.08	*0.63*
3 months after surgery	396	1.54	437	1.57	-0.03	-0.09 - 0.04	*0.42*
6 months after surgery	377	1.45	397	1.48	-0.03	-0.09 - 0.03	*0.3*
**Usual activities**							
Pre-surgery	437	1.72	466	1.75	-0.03	-0.12 - 0.07	*0.56*
3 months after surgery	396	1.98	437	1.92	0.05	-0.04 - 0.14	*0.24*
6 months after surgery	377	1.93	397	1.84	0.09	0.00 - 0.18	0.04*
**Pain/discomfort**							
Pre-surgery	437	1.91	466	1.86	0.05	-0.05 - 0.15	*0.33*
3 months after surgery	396	1.76	437	1.77	-0.01	-0.09 - 0.06	*0.74*
6 months after surgery	377	1.72	397	1.73	-0.01	-0.09 - 0.07	*0.82*
**Anxiety/depression**							
Pre-surgery	437	1.53	466	1.52	0.00	-0.08 - 0.09	*0.96*
3 months after surgery	396	1.45	437	1.42	0.02	-0.05 - 0.09	*0.49*
6 months after surgery	377	1.43	397	1.42	0.02	-0.05 - 0.09	*0.62*

Before = before implementing the rapid response system (RRS); after = after implementing the RRS; EQ-5D = Euroqol 5 dimensions, scale 1–3; 1 = no problems, 2 = some/moderate problems, 3 = severe/ extreme problems;

ASA-PS = American Society of Anesthesiologists physical status. Pre-surgery: fixed factors: gender, ASA-PS; covariates: age at admission. Following surgery: fixed factors: gender, ASA-PS; covariates: age at admission, length of stay planned intensive care admission, EQ-5D dimension pre surgery. * *p ≤ 0.05 statistical significant*.

#### Variables related with HRQOL outcomes

Table 4 shows the results for variables related to HRQOL outcomes. The pre-surgery EQ-5D index and ASA scores were significantly related to the EQ-5D index at 3 and 6 months following surgery (*p ≤ 0.01* for EQ-5D and ASA at 3 months, *p ≤ 0.01* for EQ-5D and *p = 0.02* for ASA at 6 months). Gender, age and LOS of planned ICU admissions were not significantly related with EQ-5D index scores at 3 and 6 months following surgery.

**Table 4:** Variables related with health-related quality of life outcomes

	**ASA**	**B**	**95% CI**
3 months after surgery			
Intercept		0.12	0.43 - -0.18
Before RRS implementation vs after		- 0.02	- 0.05 - 0.02
Gender, male vs female		0.02	- 0.02 - 0.05
Age		≤ 0.01	0.30 - ≤0.01
LOS planned ICU		≤ 0.01	≤ - 0.01- ≤0.01
ASA 1 tot 4 vs ASA 5	1	0.42	0.14 - 0.71
	2	0.41	0.12 - 0.69
	3	0.34	0.05 - 0.62
	4	0.37	0.06 - 0.68
EQ-5D pre surgery		0.26	0.21- 0.31
6 months after surgery			
Intercept		0.25	- 0.04 - 0.54
before RRS implementation vs after		- 0.01	- 0.05 - 0.02
Gender, male vs female		0.03	≤ - 0.01 - 0.06
Age		≤ 0.01	≤ - 0.01 - ≤0.01
LOS planned ICU		≤ 0.01	≤ - 0.01 - ≤0.01
ASA 1 to 4 vs ASA 5	1	0.32	0.05 - 0.60
	2	0.28	0.01 - 0.56
	3	0.25	- 0.02 - 0.52
	4	0.26	- 0.05 - 0.56
EQ-5D pre-surgery		0.24	0.19 - 0.29

EQ-5D = Euroqol 5 index scale -0.33 - 1; RRS = rapid response system; ASA = American Society of Anesthesiologists physical status.

LOS planned ICU = length of stay planned intensive care admission; CI = confidence interval.

## Discussion

We conclude that the implementation of an RRS does not result in a clinically relevant improvement of HRQOL as measured with the EQ-5D and EQ-VAS in patients at 3 and 6 months following major surgery. It is unlikely that the slightly less deterioration in period 2 regarding ‘mobility’ and ‘usual activities’ dimensions, which may enable patients to more actively participate in social life, can be attributed to the implementation of the RRS.

The lack of effect on HRQOL may partly be explained by the fact that our RRS was not fully mature. In particular, the MET was not consulted in 50% prior to an SAE, even though abnormal vital indicators were observed [7]. Furthermore, the percentage of included patients who experienced one or more unplanned ICU admissions in period 1 and 2 was considerably low: 2.8% and 4.5%, respectively. The number of unplanned ICU admissions could, therefore, not substantially influence the mean HRQOL scores.

Comparison of HRQOL in the subset of patients with an unplanned ICU admission also showed no improvement after RRS implementation. These results are in line with our original study on the effects of an RRS on SAEs where we showed no decrease in the Acute Physiology and Chronic Health Evaluation (APACHE) II score at admission to the ICU after RRS implementation, indicating that patients were not referred to the ICU in an earlier stage of illness [7].

Our choice to use the EQ-5D as a measure for HRQOL could be questioned, as Brazier et al. (2004) showed a ceiling effect in the EQ-5D in comparison with the short form 6 dimensions (SF-6D) instrument. This ceiling effect may partially explain the lack of effect in our study because ‘no problems’ were reported in both periods in 25% to 50% of the EQ dimensions at pre-surgery, making improvement on those scores impossible. However, Brazier et al. (2004) also showed that the SF-6D, compared to the EQ-5D, differentiates less accurately when patients experience severe health problems, which was the case for a considerable part of our study population [14]. Moreover, a comparative review of seven generic HRQOL instruments shows no uniformly ‘best’ or ‘worst’ performing instrument. The choice of the instrument should be driven by the purpose of the measurement [15]. We used the EQ-5D because the instrument is short and user friendly, which was important since a part of our study population was severely ill. The EQ-5D takes respondents about 7 minutes to complete. We believe, however, that measuring HRQOL with another generic instrument would have yielded similar results.

The most important explanation for our lack of effect is most likely that other factors had a larger influence on HRQOL than merely the implementation of an RRS. We found that pre-surgery HRQOL and ASA-PS were strongly associated with HRQOL following surgery. Similarly, another study showed that HRQOL strongly associates with diagnostic categories [16]. Associations between HRQOL and these factors may have abated the influence of the RRS implementation on HRQOL. Therefore, the question arises if HRQOL is an adequate measure to assess the influence of an RRS.

EQ-5D and EQ-VAS outcomes showed slightly different patterns. Even though the EQ-VAS scores are predictable from the EQ-5D scores, other group variables also contribute to the EQ-VAS score, such as psychological disposition, age, education and clinically-important distress. These variables explain the differences between the EQ-5D and EQ-VAS outcomes [17].

To our knowledge, this is the first study evaluating the influence of an RRS on HRQOL in patients 3 and 6 months following surgery. We conducted a cohort study before and after RRS implementation. Confounders other than the implementation of an RRS may have biased the results. However, no major changes in surgical procedures or ward policy were implemented during the study period. The pre-surgery HRQOL enabled us to study the impact of pre-admission HRQOL scores on the HRQOL at 3 and 6 months following surgery, which we considered one of the study’s strengths. One may argue that the 6-month follow-up period was too short to evaluate HRQOL improvement in surgical patients. However, improvement was most obvious during the first three months, whereas during the last three months only a slight improvement was observed. Furthermore, a longer observation period usually results in the occurrence of other confounders.

Finally, this study was conducted in one hospital and included only patients with major surgery. Results may therefore be different in other settings and with other study populations.

## Conclusions

Implementation of an RRS did not convincingly affect HRQOL outcomes. We question if HRQOL is an adequate measure to assess the influence of an RRS. Pre-surgery HRQOL and ASA-PS scores were strongly associated with HRQOL outcomes following surgery and may have abated the influence of the RRS implementation.

## Abbreviations

APACHE: Acute physiology and chronic health evaluation; ASA-PS: American Society of Anesthesiologists physical status classification; EQ-5D: EuroQol-5 dimensions; HRQOL: Health-related quality of life; ICU: Intensive care unit; LOS: Length of stay; RRS: Rapid response system; VAS: Visual analogue scale; SPSS: Statistical package for the social sciences.

## Competing interests

The authors declare that they have no competing interests.

## Authors’ contributions

FS and LS participated in the study’s design, acquisition, data management and analysis, and drafting and preparing the manuscript for publication. BF, JM and JH contributed to the study’s design, data analysis and preparing the manuscript for publication. All authors read and approved the final manuscript.

## References

[B1] DeVita MA Smith GB Adam SK Adams-Pizarro I Buist M Bellomo R Bonello R Cerchiari E Farlow B Goldsmith D Haskell H Hillman K Howell M Hravnak M Hunt EA Hvarfner A Kellett J Lighthall GK Lippert A Lippert FK Mahroof R Myers JS Rosen M Reynolds S Rotondi A Rubulotta F Winters B “Identifying the hospitalised patient in crisis” a consensus conference on the afferent limb of rapid response systems Resuscitation 2010 81 4 375 382 10.1016/j.resuscitation.2009.12.008 20149516

[B2] DeVita MA Bellomo R Hillman K Kellum J Rotondi A Teres D Auerbach A Chen WJ Duncan K Kenward G Bell M Buist M Chen J Bion J Kirby A Lighthall G Ovreveit J Braithwaite RS Gosbee J Milbrandt E Peberdy M Savitz L Young L Harvey M Galhotra S Findings of the first consensus conference on medical emergency teams Crit Care Med 2006 34 9 2463 2478 10.1097/01.CCM.0000235743.38172.6E 16878033

[B3] Esmonde L McDonnell A Ball C Waskett C Morgan R Rashidian A Bray K Adam S Harvey S Investigating the effectiveness of critical care outreach services: a systematic review Intensive Care Med 2006 32 11 1713 1721 10.1007/s00134-006-0380-6 17019547

[B4] McGaughey J Alderdice F Fowler R Kapila A Mayhew A Moutray M Outreach and Early Warning Systems (EWS) for the prevention of intensive care admission and death of critically ill adult patients on general hospital wards Cochrane Database Syst Rev 2007 3 CD005529 10.1002/14651858.CD005529.pub2 17636805

[B5] Barbetti J Lee G Medical emergency team: a review of the literature Nurs Crit Care 2008 13 2 80 85 10.1111/j.1478-5153.2007.00258.x 18289186

[B6] Jones DA DeVita MA Bellomo R Rapid-response teams N Engl J Med 2011 365 2 139 146 10.1056/NEJMra0910926 21751906

[B7] Simmes FM Schoonhoven L Mintjes J Fikkers BG van der Hoeven JG Incidence of cardiac arrests and unexpected deaths in surgical patients before and after implementation of a rapid response system Ann Intensive Care 2012 2 1 20 10.1186/2110-5820-2-20 22716308 PMC3425134

[B8] Devlin NJ Parkin D Browne J Patient-reported outcome measures in the NHS: new methods for analysing and reporting EQ-5D data Health Econ 2010 19 8 886 905 10.1002/hec.1608 20623685

[B9] Brooks R Rabin R De Charro F The measurement and valuation of health status using EQ-5D: A European perspective 2003 Kluwer Academic Publishers

[B10] Lamers LM McDonnell J Stalmeier PF Krabbe PF Busschbach JJ The Dutch tariff: results and arguments for an effective design for national EQ-5D valuation studies Health Econ 2006 15 10 1121 1132 10.1002/hec.1124 16786549

[B11] Hoeymans N Van LH Westert GP The health status of the Dutch population as assessed by the EQ-6D Qual Life Res 2005 14 3 655 663 10.1007/s11136-004-1214-z 16022059

[B12] Heyworth IT Hazell ML Linehan MF Frank TL How do common chronic conditions affect health-related quality of life? Br J Gen Pract 2009 59 568 e353 e358 10.3399/bjgp09X453990 19656444 PMC2765853

[B13] Buist M Bernard S Nguyen TV Moore G Anderson J Association between clinically abnormal observations and subsequent in-hospital mortality: a prospective study Resuscitation 2004 62 2 137 141 10.1016/j.resuscitation.2004.03.005 15294398

[B14] Brazier J Roberts J Tsuchiya A Busschbach J A comparison of the EQ-5D and SF-6D across seven patient groups Health Econ 2004 13 9 873 884 10.1002/hec.866 15362179

[B15] Whitehurst DG Bryan S Lewis M Systematic review and empirical comparison of contemporaneous EQ-5D and SF-6D group mean scores Med Decis Making 2011 31 6 E34 E44 10.1177/0272989X11421529 21993430

[B16] Badia X Diaz-Prieto A Gorriz MT Herdman M Torrado H Farrero E Cavanilles JM Using the EuroQol-5D to measure changes in quality of life 12 months after discharge from an intensive care unit Intensive Care Med 2001 27 12 1901 1907 10.1007/s00134-001-1137-x 11797026

[B17] Whynes DK Correspondence between EQ-5D health state classifications and EQ VAS scores Health Qual Life Outcomes 2008 7 6 94 18992139 10.1186/1477-7525-6-94 PMC2588564

